# A Complementary Multitechnique Approach to Assess the Bias in Molecular Weight Determination of Lignin by Derivatization-Free Gel Permeation Chromatography

**DOI:** 10.1021/acs.analchem.4c01187

**Published:** 2024-06-18

**Authors:** Daniel Papp, Göran Carlström, Tommy Nylander, Margareta Sandahl, Charlotta Turner

**Affiliations:** † Department of Chemistry, Centre for Analysis and Synthesis, Lund University, P.O. Box 124, Lund SE-22100, Sweden; ‡ Department of Chemistry, Physical Chemistry, Lund University, P.O. Box 124, Lund SE-22100, Sweden

## Abstract

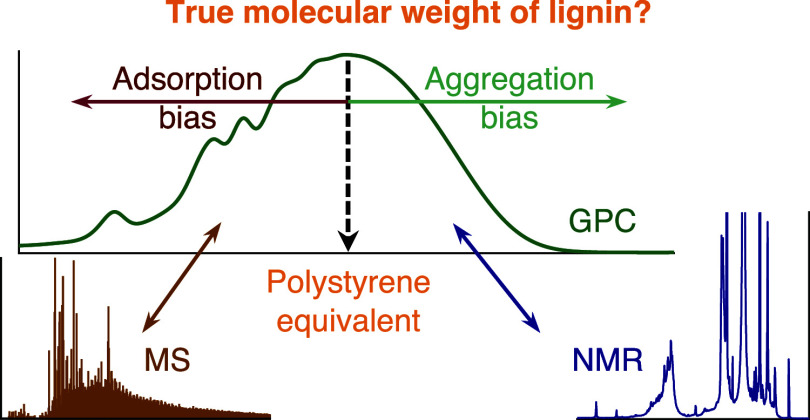

The growing interest in lignin valorization in the past decades calls for analytical techniques for lignin characterization, ranging from wet chemistry techniques to highly sophisticated chromatographic and spectroscopic methods. One of the key parameters to consider is the molecular weight profile of lignin, which is routinely determined by size-exclusion chromatography; however, this is by no means straightforward and is prone to being hampered by considerable errors. Our study expands the fundamental understanding of the bias-inducing mechanisms in gel permeation chromatography (GPC), the magnitude of error originating from using polystyrene standards for mass calibration, and an evaluation of the effects of the solvent and type of lignin on the observed bias. The developed partial least-squares (PLS) regression model for lignin-related monomers revealed that lignin is prone to association mainly via hydrogen bonding. This hypothesis was supported by functional group-based analysis of the bias as well as pulse field gradient (pfg) diffusion NMR spectroscopy of model compounds in THF-*d*_8_. Furthermore, although the lack of standards hindered drawing conclusions based on functionalities, direct infusion electrospray ionization mass spectrometry indicated that the relative bias decreases considerably for higher molecular weight species. The results from pfg-diffusion NMR spectroscopy on whole lignin samples were comparable when the same solvents were used in both experiments; in addition, the comparison between results obtained by pfg-diffusion NMR in different solvents gives some additional insights into the aggregation.

## Introduction

Lignin is, after cellulose, the second most abundant biopolymer on earth, found in most vascular plants and making up 15–40% of the plant material.^1^ While the paper and pulping industry produces about 100 million tons of lignin every year, only a few percent of this amount is utilized in biomass valorization processes.^2^ Converting lignin to value-added chemicals by various means such as reductive catalytic fractionation (RCF),^3,4^ bacterial conversion,^5,6^ and base-catalyzed depolymerization^7^ requires knowledge of basic properties such as average molecular weight (MW) and molecular weight distribution (MWD) of lignin in each step of the processes. These are crucial factors to follow during each step of the process to rationally design and improve these technologies. In industrial practice, the most widespread technique for obtaining information on the molecular weight distribution is size exclusion chromatography (SEC).

Size exclusion chromatography of lignin is predominantly performed through gel permeation chromatography (GPC)^8−10^ as most lignins are more soluble in organic solvents than in aqueous media; however, gel-filtration chromatography (GFC) methods are also available in the literature.^11,12^ Routine GPC measurements use polystyrene-divinylbenzene (PS-DVB) copolymer resins for separation with tetrahydrofuran (THF) eluent coupled with a single concentration detector, for instance, a refractive index (RI) or ultraviolet–visible (UV–vis) light absorbance detector.^13^ Conventional mass calibration, *id est* correlating the elution volume to the molecular weight via known molecular weight standards, is usually performed using linear polystyrene. To facilitate the solubilization of the lignin sample in the solvent as well as to reduce nondesirable adsorption effects, several derivatization procedures have been developed and used. These include acetylation,^14^ acetobromination,^15^ and methylation.^16^ However, Andrianova *et al.* demonstrated that the solubility of nonderivatized kraft lignins is greatly improved if THF is used in combination with water. Moreover, they found that derivatization reactions may skew the results, distorting the determined MW toward higher values.^17^ Recently, several studies reported methods employing silica-based stationary phases with more polar eluents such as DMSO and DMF using salt additives.^18,19^ Nevertheless, THF-based GPC with PS-calibration is still a commonly employed method.^20−23^

Although GPC is a well-established technique for rapid lignin analysis, the determined molecular weight of the sample is heavily dependent on the instrument setup, derivatization method, and data treatment as found by interlaboratory comparisons.^24,25^ This bias originates from the fact that currently there are no appropriate standards for lignin; instead, polystyrene (PS) is used for molecular weight calibration.^26^ Due to the obvious structural differences between lignin-related phenolic compounds and linear PS, it is likely that their respective behavior in the GPC system also differs, resulting in erroneous MW determination. To reduce these errors, for instance, multiangle laser light scattering (MALLS) can be coupled after the size exclusion column. However, MALLS suffers from multiple limitations, for instance, fluorescence activity of lignin species and UV absorption near the laser wavelength.^27^ Online coupling of electrospray ionization mass spectrometry (ESI-MS) to GPC has also been reported;^28^ however, the MS analysis is more frequently performed offline on separated lignin fractions. Nevertheless, results reported from ESI-MS measurements may also be biased due to varying ionization efficiencies and in-source fragmentation. Furthermore, the limited mass range of analyzers and complicated data analysis pose challenges.^29^ For this reason, matrix-assisted laser desorption ionization-time-of-flight (MALDI-ToF) is a more commonly used source for offline mass spectrometry.^30−32^ In addition, nuclear magnetic resonance (NMR) spectroscopy experiments have been successfully applied to directly measure the diffusion coefficient of various lignin samples.^33,34^ This technique offers a promising approach to determining molecular weight independent from the size exclusion chromatography process.

In the literature, two main sources of error have been described, which affect accurate GPC molar mass determination. The presence of non-SEC interactions between lignophenolic species and the packing material^27^ causes additional retardation of the analytes in the column and consequently an underestimation of the molecular weight. In addition, in situ formed lignin aggregates in solution lead to an overestimation of true MW.^35^ To counteract the formation of aggregates, more polar solvents such as dimethylformamide (DMF)^35^ or *N*,*N*-dimethylacetamide (DMAc)^18^ with inorganic salt additives^36^ have been suggested as eluents. Alternatively, hydroxyl groups, which are hypothesized to have a crucial role in both processes,^37^ can be derivatized.

To our knowledge, only a few studies have systematically investigated the limitations of conventional GPC calibration via modeling using known lignophenolics standards. Andrianova *et al.* explored the link between the elution volume and p*K*_a_ value for various monomers and dimers; however, they did not observe any correlation in their THF-PS-DVB GPC system.^38^ Furthermore, no other physicochemical properties of the model compounds were included in the study. The present work addresses the lack of such quantitative modeling by including more physicochemical properties and structural element-based exploration of the bias.

Focusing on the underlying mechanisms for measurement bias, we applied a complementary multitechnique approach to study a THF-based system with a PS-DVB column, shown^13^ to be one of the most widely employed methods to determine the MW distribution of kraft and organosolv lignins. Traditionally, samples are derivatized prior to THF-based GPC; nevertheless, recent works of Andrianova *et al.*(17) and LaVallie *et al.*(23) proved that this step can be omitted. The main aim of our work is to assess the bias of this simplified method, which does not include derivatization, thus being greener and easier to apply. The errors were estimated by the comparison of determined and true molecular weights of a set of monomer and dimer lignin model compounds. A partial least-squares (PLS) regression model was developed to link physicochemical properties to the observed bias of the model compounds. In addition, relatively narrow molecular weight distribution kraft lignin fractions were analyzed by direct infusion ESI-MS to evaluate the bias in the higher MW ranges as well. To decouple the adsorption effects from aggregation as well as to circumvent the low optical contrast between analyte and solvent, limiting the feasibility of light scattering methods, pulsed field gradient diffusion NMR (pfg-diffusion NMR) studies were conducted to confirm the findings of the previous experiments. While none of these techniques are free from measurement bias *per se*, the different orthogonal aspects and the combination of results provided by them offer a deeper insight into the behavior of lignin in a gel permeation system.

## Experimental Section

### Chemicals

Tetrahydrofuran (THF), acetone, and methanol (MeOH) were all of HiPerSolv ChromaNorm quality and purchased from VWR International (Radnor, PA, USA). Dimethyl sulfoxide-*d*_6_ (DMSO-*d*_6_), THF-*d*_8_, *N*,*N*-dimethylformamide-*d*_7_ (DMF-*d*_7_), and 2 M ammonia in methanol were purchased from Sigma-Aldrich (St Louis, MO, USA). The water used in the experiments was purified in-house with a Merck Millipore water purification system (Millipore, Billerica, MA, USA).

Lignin-related phenolic model compounds (listed in SI Table S1) were prepared as single compound standards in methanol with a concentration of 1 mg/mL. Altogether, 32 monomers and seven dimers were used.

The investigated lignin samples involved an organosolv lignin (provided by Ola Wallberg, Lund University, Sweden), an Indulin AT kraft lignin (Omar Abdelaziz, Lund University, Sweden), and a birch sawdust oil from reductive catalytic fractionation (Joseph Samec, Stockholm University, Sweden).

### Gel Permeation Chromatography

GPC was performed on an Agilent 1100 system (Agilent Technologies, Santa Clara, CA, USA), consisting of a G1313A autosampler, a G1311A quaternary pump, and a G1314A variable wavelength detector. Column temperature was regulated by a Model 7955 column thermostat (Jones Chromatography, Hengoed, UK). The instrument was controlled by Openlab Chemstation Edition Version 3.5 software. Initial GPC experiments exhibited an excellent reproducibility of the determined MW (RSD < 1%, data not shown); thus, the random error originating from reproducibility was assumed to be negligible. For this reason, GPC results throughout the manuscript are presented as single measurement results.

Separation was conducted on an Agilent PLGel column (Agilent Technologies, Santa Clara, CA, USA) with the corresponding guard column, both thermostated at 50 °C. The length, diameter of the column, and average pore size of the packing were 300 mm, 7.5 mm, and 500 Å, respectively. The flow rate of the eluent was set to 1 mL/min, and detection was carried out at 254 nm. Linear polystyrene standards in the range of 162–20000 Da (Agilent EasiVial PS-L) were used for mass calibration (SI Figure S1). The injection volumes were 10 μL for the lignin model compounds and 20 μL for the fraction collection. Relative error for the lignin model compounds was calculated according to eq 1, where MW_observed_ is the molecular weight calculated from the mass calibration of the column by linear PS standards.
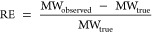
1

A partial least-squares (PLS) regression model to predict the relative error from molecular descriptors was developed using MATLAB Version R2021b and using a freely available regression toolbox.^39^ Molecular descriptors were predicted by the commercially available Hansen Solubility Parameters in Practice software and used as independent variables. To ensure that the model yields valid predictions for the whole range, model compounds were manually split into training and test subsets to cover the whole response range instead of using random splitting. Variable selection was conducted by means of a genetic algorithm (500 runs) followed by forward selection.

### Fractionation and Direct Infusion Mass Spectrometry Analysis of Indulin AT Lignin

The Indulin AT lignin sample was chosen for fractionation and subsequent MS analysis due to its wide MW distribution. Using the described method above, narrow MW fractions were collected from the GPC effluent using a Model 704 fraction collector (Varian, Palo Alto, CA, USA) to perform a time-based collection of fractions (Supporting Information Table S2). Altogether the samples from 85 runs were pooled, dried under nitrogen stream, and then reconstituted in acetone:water:5 mM ammonia in MeOH (35:15:50 V/V/V%) solvent prior to direct infusion to the mass spectrometer.

Direct infusion ESI-MS experiments were conducted on a Xevo G2 QToF-MS (Waters, Milford, MA) with a 74900 series syringe pump (Cole-Parmer, Vernon Hills, IL, USA) used for infusion of the sample. Pooled Indulin AT kraft lignin fractions were infused with a 0.4 mL/h flow rate. The MS acquisition parameters were optimized manually to ensure soft ionization and minimize in-source fragmentation. ESI was performed in negative mode with the capillary voltage set to 2.5 kV, sampling cone voltage to 35 V, source temperature at 120 °C, desolvation temperature at 300 °C, and cone gas and desolvation gas at 40 and 400 L/h, respectively. High-resolution mass spectra were collected with a scan rate of 1 scan/s for 2 min in the range of 50–1200 *m*/*z* for fractions FR I–FR III, and the 100–2000 *m*/*z* range was used for fractions with a higher expected average mass. The quadrupole profiles were manually tuned before each acquisition to yield the best possible sensitivity.

ESI-MS spectra were blank-corrected, and the number-averaged molecular weights of the fractions were computed according to eq 2, where *I*_*i*_, *z*_*i*_, and (*m*/*z*)_*i*_ are the intensity, charge, and mass-to-charge ratio of the *i*^th^ ion, respectively
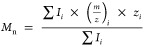
2

### NMR Experiments

NMR experiments were conducted at 25 °C on a Bruker Avance III HD 500 MHz spectrometer (Bruker, Billerica, MA, USA) equipped with a room temperature 5 mm BBFO SmartProbe. Pulsed field gradient diffusion NMR measurements were performed using a stimulated echo, bipolar gradient pulses, a longitudinal eddy current delay (LED), and two spoil gradient pulses. The pulse sequence used was ledbpgp2s from the Bruker standard library. The gradient strength was increased linearly between 2 and 98% of the maximum field strength of 48.2 G/cm in 32 steps. The field strength was calibrated by diffusion measurements of trace amounts of HDO in D_2_O and adjusting the maximum gradient amplitude to achieve a diffusion constant of 1.902 m^2^/s at 25 °C.^40^ The lengths of the encoding and decoding gradient pulses (∂) were 2 ms and had a smooth rectangular shape (SMSQ10.100). The diffusion time (Δ) was kept constant at 200 ms for the lignin samples, while 50 ms was kept for the field strength calibration experiment. The LED was 5 ms, and the length of the spoil gradient pulses was 600 μs, applied with an amplitude of −17.13 and −13.17% of the maximum gradient strength. The acquisition time used was 1 s, and the recycle time was 4 s. Typically, 32 scans were acquired for each gradient strength after 16 dummy scans. Exact temperature calibration of the instrument was based on the ^1^H spectrum of pure methanol, using the distance between the two peaks as described by Ammann *et al.*(41)

*p*-Hydroxybenzoic acid, vanillic acid, syringic acid, *p*-hydroxybenzaldehyde, vanillin, syringaldehyde, *p*-hydroxybenzylalcohol, vanillyl alcohol, and syringyl alcohol were prepared in THF-*d*_8_ with 10 mg/mL concentration. Indulin AT kraft lignin, organosolv lignin, and birch lignin oil from RCF samples were prepared in THF-*d*_8_, DMSO-*d*_6_, and DMF-*d*_7_ to concentrations of 10 mg/mL. All experiments were run in triplicates.

All NMR data were processed and analyzed by the freely available GNAT toolbox^42^ for MATLAB.^43^ The workflow included Fourier transformation of the raw data, after multiplication with an exponential window function (1 Hz) and zero filling to 32,000 data points, as well as phase and linear baseline correction. The decay curves were fitted to the Stejskal–Tanner equation,^44^ modified to account for the effects from the gradient shape. In the corrected spectra, the aromatic region of the lignin species between 6.5 and 8 ppm (see the spectra in SI Figure S2) was integrated and used to find the diffusion constant. This was converted to molecular weight using the Diffusion Estimation module of the GNAT toolbox, which estimated the necessary parameters using solvent and temperature data as input. The Stokes–Einstein–Gierer–Wirtz equation (SEGWE) was used by the software for calculations, which describes the diffusion of small molecules more accurately than the classical Stokes–Einstein equation. For further discussion of the superiority of SEGWE over the classical Stokes–Einstein relationship for small molecular diffusion estimation by NMR, the reader is referred to the study by Evans et al.^45^
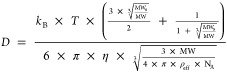
3

## Results and Discussion

### GPC Molecular Weight Determination Using Lignin Model Compounds—Modeling and Error Analysis

**Figure 1:**
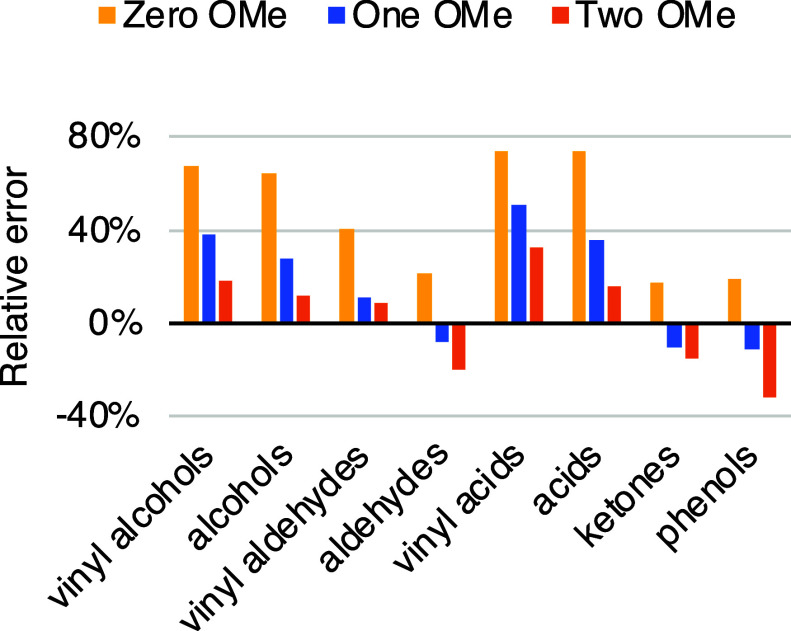
Relative error of MW determination of lignin monomers grouped according to their functionalities. The graph highlights the effect of the number of methoxy groups (OMe) on the monomers.

Quantitative estimate for the bias in the molecular weight determination was obtained by a thorough analysis of 39 lignin model compounds involving monomers and dimers. This was carried out by running the model compounds in the GPC system and calculating their molecular weights using the mass calibration curve established with linear PS standards (SI Figure S1). Errors of the determined MW of lignin standards, as presented in Figure 1, ranged between −47 and 73%, indicating considerable disagreement between the true and determined molecular weights of the model compounds. It should be noted that at this low MW range, a substantial regression error can be expected, which contributes to the error.

**Figure 2:**
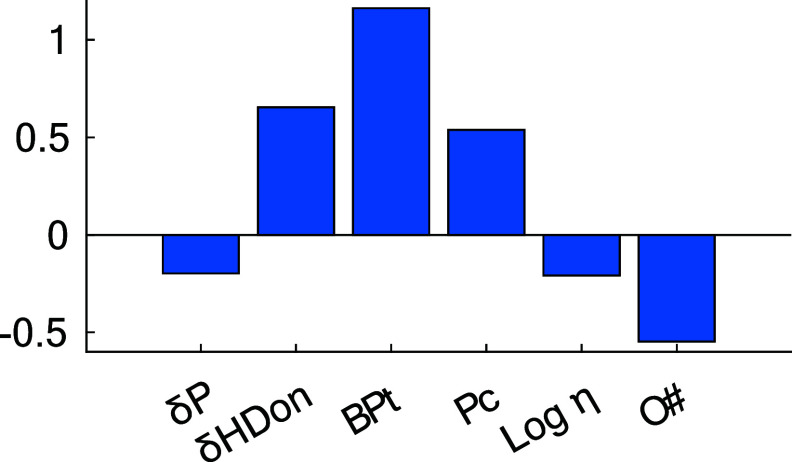
Standardized coefficients of variables in the PLS model. δP and δH_Don_ are Hansen solubility parameters^49^ describing the capability to establish polar and proton donating interactions. BPt: boiling point. *P*_c_: critical pressure. Log η is the logarithm of viscosity. O#: number of oxygen atoms.

To establish a relationship between certain molecular descriptors and the observed relative error, a PLS regression model was developed with the relative error of the MW determination being the dependent variable. The developed model featured four latent variables that were able to explain 94% of the variance in the data. The coefficient of determination values for the training and test data set were 0.818 and 0.751, while root-mean-square errors were 10.6 and 12.4, respectively. The performance of the developed model was evaluated on a test set of seven model compounds to predict the relative error against polystyrene. As shown in Table 1, the accuracy of prediction on the test set is satisfactory, despite the apparently low *R*^2^ values. Standardized coefficients of the selected variables are also presented in Figure 2.

**Table 1:** Results of the Prediction of the Relative Bias for Compounds in the Test Set^a^

analyte	true MW (g/mol)	observed relative error (%)	predicted relative error (%)
coniferyl alcohol	180.2	38.6	37.8
*p*-coumaric acid	164.2	73.8	79.2
D3	274.3	26.0	21.4
syringaresinol	418.4	4.0	5.0
isoeugenol	164.2	–5.5	–2.4
M6	212.2	21.2	26.3
acetosyringone	196.2	–14.3	–10.6

a The observed relative error is calculated according to eq 1, against linear polystyrene standards. The predicted relative error is predicted by the developed PLS regression model.

Although the number of model compounds was limited, some correlations of the error with the structure and properties can be observed. As shown in Figure 2, the hydrogen bond-donating capability and boiling point are the two most important (and correlated) properties. Combining this with the large positive bias for lignin model compounds with acidic or alcohol functionalities in Figure 1, hydrogen bonds appear to be particularly important in the aggregation process. Furthermore, Figure 1 shows that the number of methoxy groups in lignin monomers is inversely related to the observed relative bias, possibly because methoxy groups act as electron donors, decreasing the acidity of the hydroxyl groups. At the same time, negative bias was found in the case of a few model compounds, implying interactions with the column packing material. Such interactions are seemingly more prominent with compounds possessing one or two methoxy groups on the phenyl group, suggesting that hydrophobicity plays a role in the mechanism.

### Direct Infusion Mass Spectrometry of Isolated Lignin Fractions

While a similar modeling for larger lignin species would be extremely useful in further investigations, due to the lack of standards, it is not possible. For this reason, if one is to investigate the bias in GPC, the method has to be benchmarked against another orthogonal technique such as mass spectrometry. To cover a wide MW range, pooled Indulin AT lignin fractions were analyzed by direct infusion mass spectrometry to obtain the molecular weight of the fractions for comparison (for the time windows, see SI Table S2). While orthogonal to GPC, MS is not without bias itself either. First of all, varying ionization efficiencies between lignin species can distort the results. Regrettably, a study on ionization efficiencies of larger lignin oligomers also requires standards; thus, it relies on in-house synthesis of model compounds. Andrianova et al. investigated seven dimeric species trying to relate various functionalities to ionization efficiency, reporting similar results for the tested analytes.^29^ Although experimental evidence has not yet been shown, we could assume that no major difference in ionization efficiency among species occurs in the higher MW fractions.

**Figure 3:**
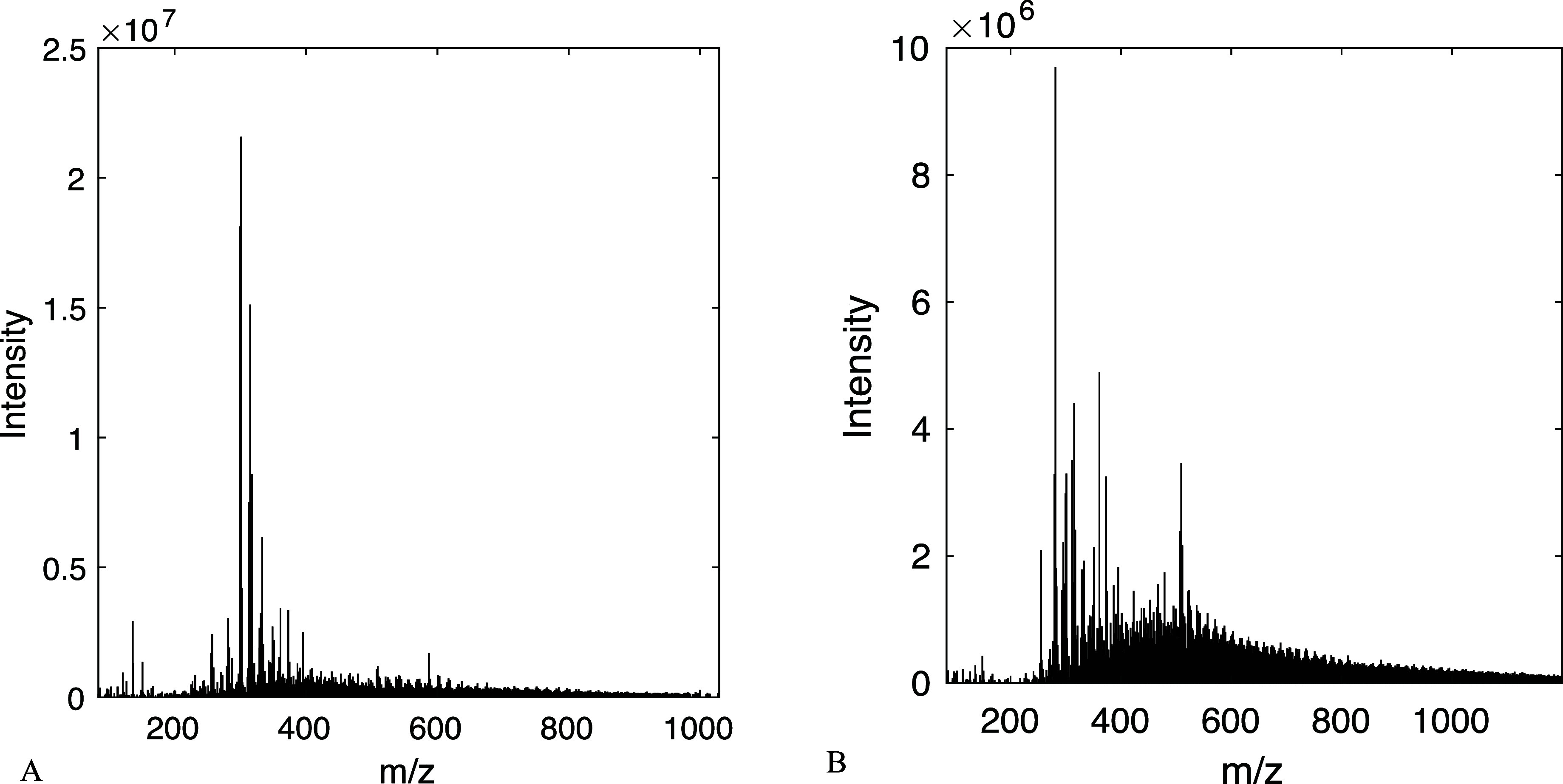
Direct infusion ESI-QToF-MS mass spectra of Indulin AT kraft lignin fractions FR I (A) and FR II (B).

Another source of error in direct infusion MS is in-source fragmentation, which would cause an underestimation of the molecular weight. During the experimental work, the source parameters were carefully optimized to avoid fragmentation. The results of this can be seen in the mass spectrum of a lignin dimer in SI Figure S3, where about 90% of the useful signal is related to the deprotonated molecular ion, corroborating with the results reported by Önnerud et al.^46^ Similar to the ionization efficiency problem, we were not able to verify our observations for higher MW species without the necessary reference materials; nevertheless, in-source fragmentation was assumed to be negligible for all fractions. With these two assumptions, the number averaged *m*/*z* of each fraction was calculated with eq 2 and presented in Table 2. The mass spectra of FR I and FR II are shown in Figure 3, while the mass spectra of the rest of the fractions are found in SI Figure S4.

**Table 2:** Comparison of Number-Averaged Molecular Weights of Indulin AT Lignin Fractions as Determined by GPC (PS-Equivalents) and Direct Infusion ESI-MS^a^

GPC fraction	GPC *M*_n_, determined directly after fractionation (Da)	GPC *M*_n_, determined after 1 month of storage (Da)	ESI-MS average *m*/*z*, determined after 1 month of storage (*m*/*z)*
FR I	162	424	454
FR II	370	578	567
FR III	580	789	696
FR IV	1000	1679	1171
FR V	2000	2919	1165
FR VI	3000	3816	1192

a The average molecular weights of the fractions were determined both directly after fractionation and after 1 month of storage at −20 °C. GPC chromatograms of the fractions are shown in the Supporting Information, Figure S5. Average *m*/*z* was measured on the 1 month-old stored samples by direct infusion ESI-MS and calculated according to eq 2.

The spectra in Figure 3 reveal a not unexpected but not yet reported phenomenon. First, the centers of the spectra of FR I and FR II are shifted in comparison to the expected average MW of the respective fractions based on GPC, determined right after fractionation using the mass calibration curve of PS. Also, instead of a Gaussian curve, the shape of the spectrum is closer to log-normal. This can possibly be traced back to repolymerization of the samples during a few weeks of storage between the fractionation and the actual MS analysis time. This hypothesis is confirmed by comparing the molecular weights of the same samples determined fresh and after 1 month of storage (Table 2). The difference varies over the MW range; the increase is the most prominent for FR I (monomers), which became more than 2.5 times larger upon storage, while the average MW of the largest fraction (FR VI) only increased by 27%. This suggests that monomers and other smaller oligomers are more susceptible to repolymerization than larger lignin species. The hypothesis regarding repolymerization is further supported by the shape of the spectrum in Figure 3B, implying that the process does not stop when two dimers are combined but the reaction goes on to form higher MW species.

Comparing the determined molecular weights from GPC and direct infusion MS in Table 2 (two rightmost columns), good agreement can be observed for the first three fractions with a gradual increase in discrepancy for the high MW fractions. Similar differences for those molecular weights were reported by Andrianova et al.^38^ Most likely, the reason for this disagreement is the formation of a multiply charged species. This is also the probable reason FR IV–FR VI have similar average *m*/*z* values. While this may prove useful for better detectability of large lignophenolics up to 7000 Da, charge state assignment and deconvolution are necessary to obtain the molecular weight of these analytes.^29^ Regrettably, the signal intensity was insufficient in our case to perform this. On the other hand, Jacobs et al. showed that results from MS and GPC do not differ substantially in the higher MW region,^47^ thus complementing our data. Overall, combining our results with those from the literature suggests that there is no major difference between the results from GPC and MS in the higher MW region. This implies that the hydrogen bond-driven aggregation, shown for monomers by PLS regression, is less prominent for larger species. A reason for this may be that as the size of the molecule increases, hydroxyl groups might be sterically shielded from establishing such interactions.

### Investigation of the Aggregation Behavior of Lignin in Common GPC Solvents by pfg-Diffusion NMR Spectroscopy

**Figure 4:**
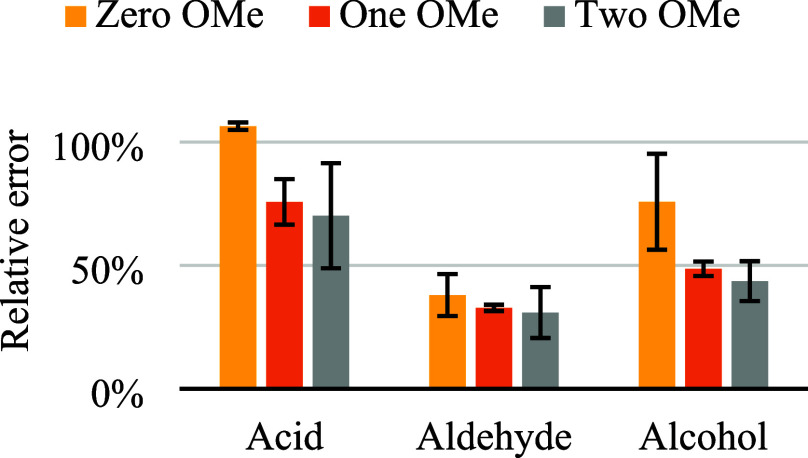
Relative error of molecular weight of selected lignophenolic model compounds in THF-*d*_8_ as determined by pfg-diffusion NMR. Error bars represent a one standard deviation range (*n* = 3).

With the intent to further investigate our hypothesis regarding the importance of association via hydrogen bonds, we determined the apparent hydrodynamic radius of nine selected lignin monomers by pfg-diffusion NMR experiments. It is important to point out that these monomers are different from those in the PLS regression model test set since in this experiment, the focus was on the systematic investigation of aggregation based on structural elements. For this reason, monomers with carboxylic acid, aldehyde, and alcohol functionalities were chosen from each monomer type (H, G, and S). The obtained relative errors, presented in Figure 4, agree with the outcomes of the GPC results, *id est* lignophenolics with an additional free hydroxyl group exhibit larger biases than the aldehydes. Furthermore, although the trend is less pronounced than in Figure 1, it also appears as the methoxy groups weaken the aggregation process in THF. This is apparent from the decrease in bias as the analyte contains more methoxy groups. On the other hand, the observed relative errors per NMR are considerably larger than those found by GPC, which is possibly explained by the fact that the effect of adsorption to the column packing material, causing an apparent negative bias in GPC, is absent in NMR.

**Figure 5:**
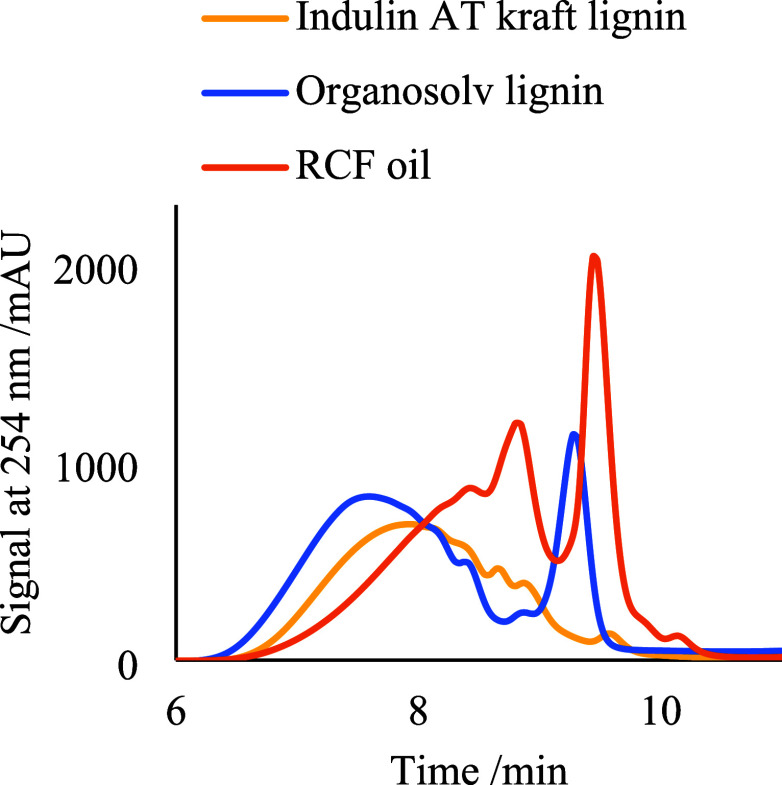
GPC chromatograms of the Indulin AT, organosolv lignin, and birch RCF sawdust oil samples. Chromatographic conditions: eluent: 1 mL/min THF; column: PLGel 500 Å column (300 × 7.5 mm) at 50 °C; detection: UV at 254 nm. Samples were diluted in THF solvent prior to injection.

While the proposed aggregation phenomenon is apparent for lignin monomers, shown by the high relative errors, technical, nondepolymerized lignins contain only a small fraction of such low-MW compounds. This can be observed in Figure 5, where a considerably higher amount of lignin monomers (large peak after 9 min) is detected in the processed birch RCF oil compared to the Indulin AT sample. Due to the relatively higher content of monomers, which are prone to more excessive aggregation, it is expected that the relative difference between GPC and NMR determinations should be higher for the birch RCF oil sample.

**Figure 6:**
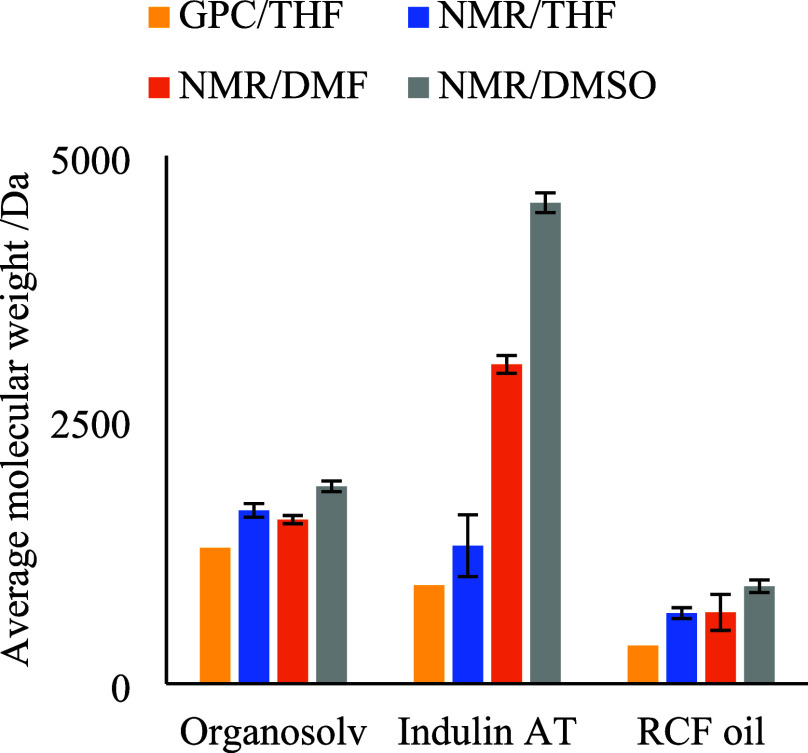
Comparison of number-averaged molecular weights by different techniques (pfg-diffusion NMR/solvent type and GPC/solvent type) for three lignin samples. Error bars represent one standard deviation (*n* = 3). GPC results, presented as single data points, were yielded by a conventionally calibrated GPC process and expressed as PS-equivalent. RSD of the replicates of the GPC experiments is below 1% (data not shown).

Aiming to investigate the comparability between GPC and NMR results, lignin samples in commonly used GPC solvents (THF, DMF, and DMSO) were analyzed by pfg-diffusion NMR. The average molecular weights were obtained from the Stokes–Einstein–Gierer–Wirtz equation (eq 3). The results were compared to those obtained by GPC as described in the Experimental Section. Figure 6 shows that when using THF as both GPC and NMR solvent (yellow and blue columns), results from the two techniques are comparable with slightly higher observed molecular weights *per* NMR. This is attributed to the occurrence of analyte–packing material interactions in GPC, which are lacking in NMR, as already suggested for the monomer model compounds. Furthermore, although its effect is considered to be less prominent, dilution of the sample in the GPC column may also impede association, thus lowering the apparent molecular weight compared to NMR. The differences between NMR and GPC results were similar across the different types of samples in the magnitude of 300–400 Da, which implies that the lignin source or extraction technology does not have a major influence on the magnitude of disagreement between the two techniques. It is worth noting, however, that in the case of depolymerized lignin samples, direct comparison of NMR and GPC might lead to erroneous conclusions such as in the case of the RCF oil sample, where the molecular weight by NMR was found to be nearly twice as high as by GPC.

Figure 6 reveals additional insights into the aggregation process in different solvents as well. Comparing the determined MW in THF, DMF, and DMSO (blue, orange, and gray columns, respectively) by pfg-diffusion NMR, there is considerable disagreement in the case of the Indulin AT sample, while the results for the other two samples corroborate better. A possible explanation for this is the different composition of the samples. Constant et al.^48^ demonstrated by quantitative ^31^P NMR that Indulin AT contains more aliphatic hydroxyl groups and carboxylic acid groups than organosolv lignins. Such functional groups were also found to be highly influential in the error of MW determination in our investigation with monomer and dimer standards. Although the results of the developed PLS regression model cannot be quantitatively extrapolated to higher molecular weights, we believe that qualitatively, the model is still valid. Similar results were concluded in a study by Zhao et al. using small angle neutron scattering, having found a relationship between the amount of aliphatic hydroxyl groups and aggregation in DMSO.^37^ This aggregation phenomenon is possibly further amplified by the fact that we did not use salt additives in the NMR experiments to disrupt aggregates; however, this is essential when DMSO or DMF are used as GPC solvents. It is important to note that we did not observe complications with dissolving our lignin samples in any of the solvents used during sample preparation, and thus, the observed differences in MW are not caused by precipitation of larger lignin compounds.

## Conclusions

Gel permeation chromatography has been the tool to rapidly determine average molecular weight and molecular weight distribution of lignin samples for decades; however, the trueness of the method has not been thoroughly investigated until very recently. In our study, a PLS regression model was developed to quantitatively describe the relationship between various physicochemical properties and the bias of molecular weight determination for lignin monomers and dimers. Such a model, to our best knowledge, has not been available before; however, its quantitative results are limited to monomers and dimers. Our PLS model and diffusion NMR suggest that intermolecular hydrogen bonds have a key role in aggregation. Thus, the error in the molecular weight determination will be the largest for phenolics with acid or alcohol functionalities. This phenomenon needs to be especially considered when analyzing depolymerized lignin samples, which contain relatively more monomers and dimers compared to technical lignins. To assess the error in the higher molecular weight range, isolated, narrow molecular weight lignin fractions were analyzed by GPC and direct infusion ESI-MS. However, in contrast to the low-MW model compounds, where large relative errors were found with GPC, the results from the two techniques showed good agreement up to the point where the occurrence of multiple charged species caused an increasing discrepancy. This indicates that the suggested aggregation process is less prominent for larger oligomers, which prompts further investigations with larger oligomer model compounds. A possible explanation for this is that although the nature of the intermolecular interactions remains the same in the case of large oligomers, other effects such as conformation also have an influence on the magnitude of interactions. In addition, with the increase in size of the molecule, the molecular surface area available for reversed-phase interactions with the column packing also increases, which counteracts the effects of aggregation. Furthermore, direct experimental evidence on the repolymerization of lignin in solution is provided. Good agreement was found between the pfg-diffusion NMR and GPC results when the same solvent was used for both analyses. Our results combined with findings published in the literature regarding the agreement between GPC and mass spectrometry^29^ as well as GPC and pfg-diffusion NMR^34^ prove that THF-based GPC is in general a reliable method for MW determination of whole lignin samples even without derivatization prior to analysis. However, GPC results might be skewed when samples with a high monomer content are investigated.

## Supporting Information Available

List of the lignin model compounds used to train and test the PLS regression model, mass calibration curve of the GPC column, time windows for the fractionation of the Indulin AT kraft lignin, ^1^H NMR spectra of various lignin samples in THF-*d*_8_ as well as mass spectra of a lignin dimer and the 1 month-old stored lignin fractions (PDF)
