# Supplementary material for: A Complementary Multitechnique Approach to Assess the Bias in Molecular Weight Determination of Lignin by Derivatization-Free Gel Permeation Chromatography
Source: Anal Chem. 2024 Jun 18;96(26):10612–9. doi: 10.1021/acs.analchem.4c01187 (PMC11223100; doi:10.1021/acs.analchem.4c01187)

Supporting information: A complementary multi-technique approach to assess the bias in molecular weight determination of lignin by derivatization-free gel permeation chromatography

Daniel Papp[†], Göran Carlström[†], Tommy Nylander[‡], Margareta Sandahl[†], Charlotta Turner*[†]

[†]Lund University, Department of Chemistry, Centre for Analysis and Synthesis, P.O. Box 124, SE-22100 Lund, Sweden

[‡]Lund University, Department of Chemistry, Physical Chemistry, P.O. Box 124, SE-22100 Lund, Sweden

TABLE OF CONTENTS

Table S1: List of lignin model compounds used for training and testing the PLS regression model S-2
Figure S1: Mass calibration curve of the PLGel 500 Å column S-6
Table S2: Time windows for the fractionation of Indulin AT kraft lignin S-7
Figure S2: 1H-NMR spectra of the investigated lignin samples S-8
Figure S3: ESI-MS spectrum of dehydrodidihydroeugenol (D1) S-10
Figure S4: Direct infusion electrospray ionization mass spectra of isolated lignin fractions S-11
Figure S5: Gel permeation chromatograms of the isolated lignin fractions after one month of storage S-14

Table S1: List of lignin model compounds used for training and testing the PLS regression model

Name	Structure	Supplier	Functional group/linkage motif
p-coumaric acid	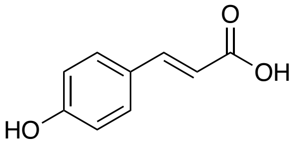	Sigma-Aldrich St Louis, MO, USA	Acid
Ferulic acid	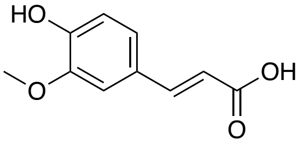	Sigma-Aldrich St Louis, MO, USA	Acid
Sinapinic acid	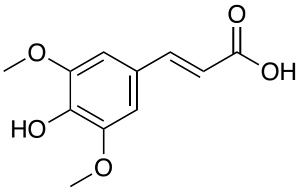	Sigma-Aldrich St Louis, MO, USA	Acid
p-hydroxybenzoic acid	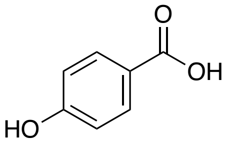	Sigma-Aldrich St Louis, MO, USA	Acid
Vanillic acid	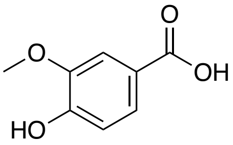	Sigma-Aldrich St Louis, MO, USA	Acid
Syringic acid	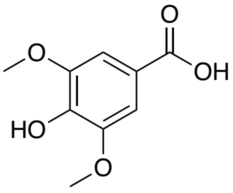	Sigma-Aldrich St Louis, MO, USA	Acid
p-coumaryl alcohol	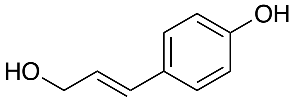	Sigma-Aldrich St Louis, MO, USA	Alcohol
Coniferyl alcohol	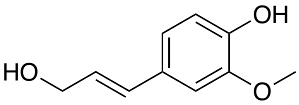	Sigma-Aldrich St Louis, MO, USA	Alcohol
Sinapyl alcohol	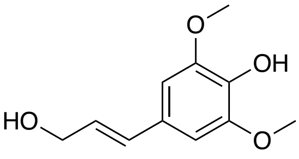	Sigma-Aldrich St Louis, MO, USA	Alcohol
p-hydroxybenzylalcohol	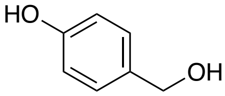	Sigma-Aldrich St Louis, MO, USA	Alcohol
Vanillyl alcohol	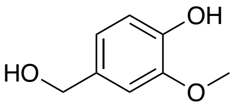	Sigma-Aldrich St Louis, MO, USA	Alcohol

Syringyl alcohol	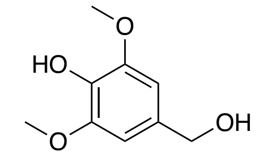	Sigma-Aldrich<br>St Louis, MO, USA	Alcohol
Coniferyl aldehyde	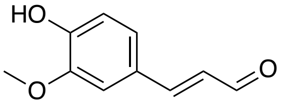	Sigma-Aldrich<br>St Louis, MO, USA	Aldehyde
Sinapaldehyde	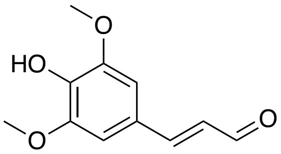	Sigma-Aldrich<br>St Louis, MO, USA	Aldehyde
p-hydroxybenzaldehyde	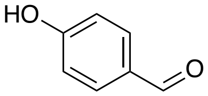	Sigma-Aldrich<br>St Louis, MO, USA	Aldehyde
Vanillin	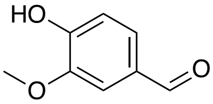	Sigma-Aldrich<br>St Louis, MO, USA	Aldehyde
Syringaldehyde	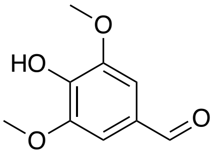	Sigma-Aldrich<br>St Louis, MO, USA	Aldehyde
Phenol	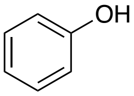	Sigma-Aldrich<br>St Louis, MO, USA	Phenol
Guaiacol	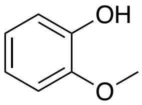	Sigma-Aldrich<br>St Louis, MO, USA	Phenol
Syringol	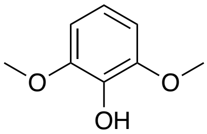	Sigma-Aldrich<br>St Louis, MO, USA	Phenol
p-hydroxyacetophenone	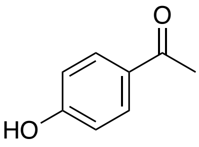	Sigma-Aldrich<br>St Louis, MO, USA	Ketone
Acetovanillone	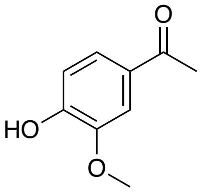	Sigma-Aldrich<br>St Louis, MO, USA	Ketone
Acetosyringone	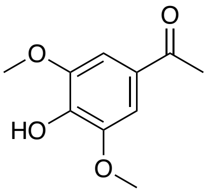	Sigma-Aldrich<br>St Louis, MO, USA	Ketone

Guaiacylglycerol-beta-guaiacyl ether	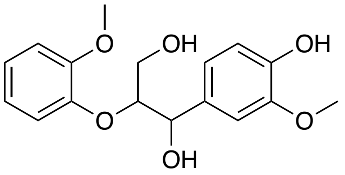	Sigma-Aldrich St Louis, MO, USA	β-O-4
Dehydrodidihydroeugenol D1	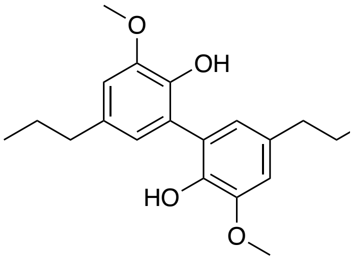	J.S.M. Samec, Stockholm University, Stockholm, Sweden	5-5’
4-[2-(4-hydroxy-3,5-dimethoxyphenyl)ethyl]-2,6-dimethoxyphenol D2	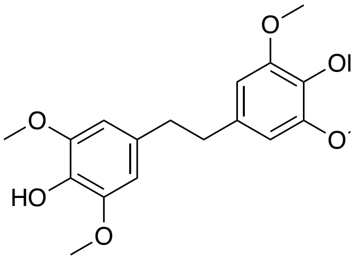	J.S.M. Samec, Stockholm University, Stockholm, Sweden	β-1
4,4'-(Ethane-1,2-diyl)bis(2-methoxyphenol) D3	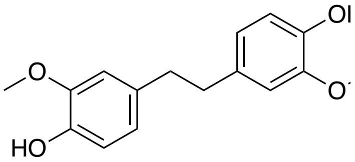	J.S.M. Samec, Stockholm University, Stockholm, Sweden	β-1
4-(4-hydroxy-3-methoxyphenethyl)-2,6-dimethoxyphenol D4	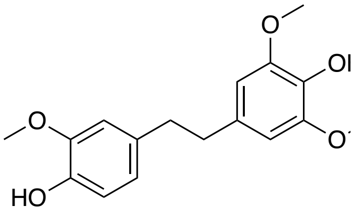	J.S.M. Samec, Stockholm University, Stockholm, Sweden	β-1
Pinoresinol D6	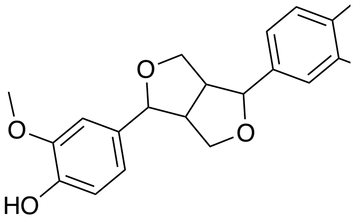	J.S.M. Samec, Stockholm University, Stockholm, Sweden	β-β
Syringaresinol D7	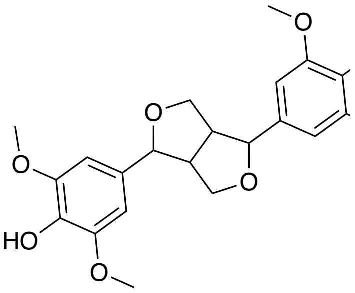	J.S.M. Samec, Stockholm University, Stockholm, Sweden	β-β

(E)-4-(3-(hydroxymethyl)-5-(3-hydroxyprop-1-en-1-yl)-7-methoxy-2,3-dihydrobenzofuran-2-yl)-2-methoxyphenol<br><br>D8	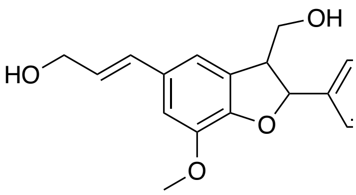	J.S.M. Samec, Stockholm University, Stockholm, Sweden	β-5
4,4'-(1-methoxyethane-1,2-diyl)bis(2,6-dimethoxyphenol<br><br>D9	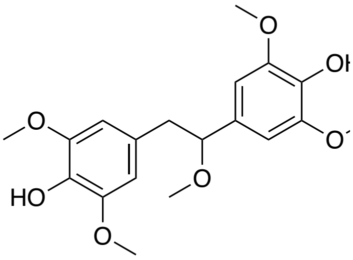	J.S.M. Samec, Stockholm University, Stockholm, Sweden	
1-(4-hydroxy-3-methoxyphenyl)-2-(2-methoxyphenoxy)propane-1,3-diol<br><br>D10	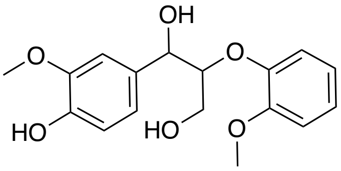	J.S.M. Samec, Stockholm University, Stockholm, Sweden	
Isoeugenol<br><br>M1	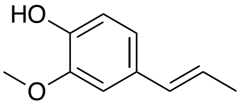	J.S.M. Samec, Stockholm University, Stockholm, Sweden	
2-methoxy-4-propylphenol<br><br>M2	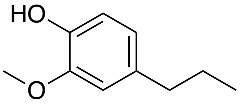	J.S.M. Samec, Stockholm University, Stockholm, Sweden	
Dihydroconiferyl alcohol<br><br>M3	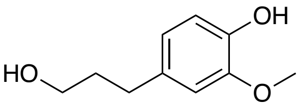	J.S.M. Samec, Stockholm University, Stockholm, Sweden	Alcohol
(E)-2,6-dimethoxy-4-(prop-1-en-1-yl)phenol<br><br>M4	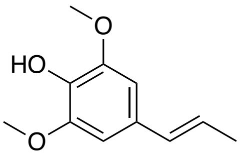	J.S.M. Samec, Stockholm University, Stockholm, Sweden	
2,6-dimethoxy-4-propylphenol<br><br>M5	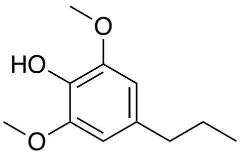	J.S.M. Samec, Stockholm University, Stockholm, Sweden	
4-(3-hydroxypropyl)-2,6-dimethoxyphenol<br><br>M6	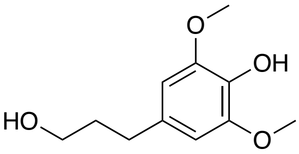	J.S.M. Samec, Stockholm University, Stockholm, Sweden	Alcohol

Figure S1: Mass calibration curve of the PLGel 500 Å column

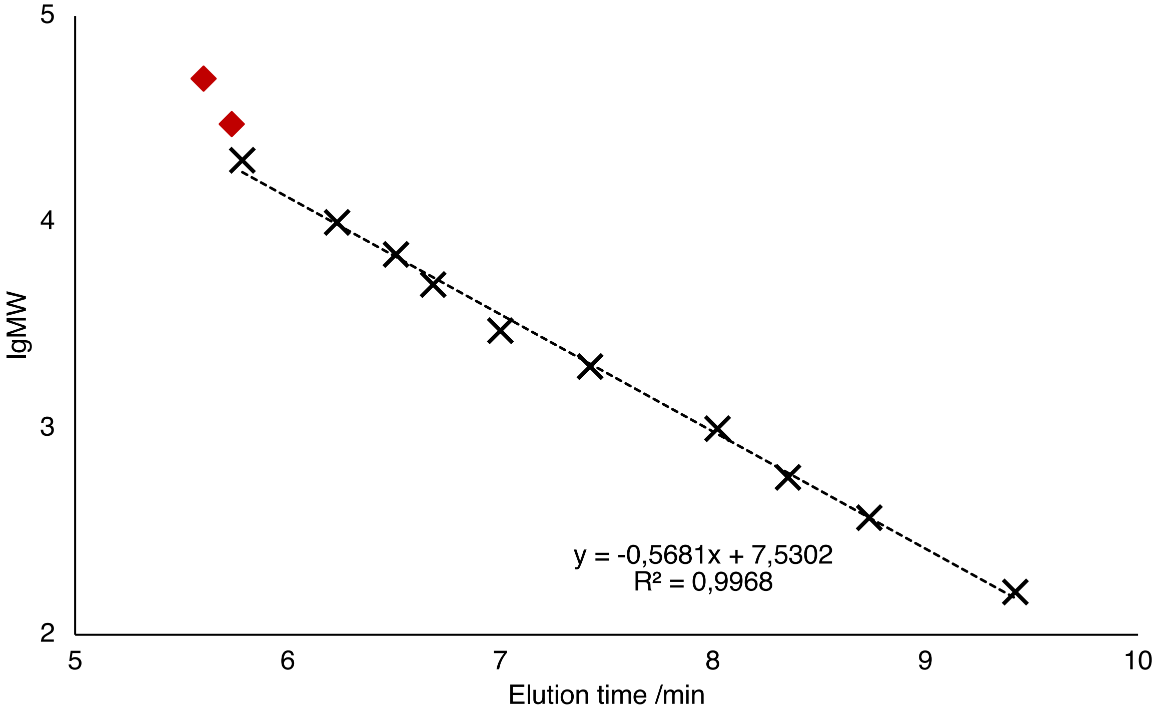

Figure S1: Mass calibration curve of the PLGel 500 Å column. Eluent: 1 mL/min THF; temperature: 50°C. Black crosses represent linear polystyrene calibrants which constitute the linear range of the column, while red diamonds denote points outside of the linear range.

Table S2: Time windows for the fractionation of Indulin AT kraft lignin

Fraction number	Fraction molecular weight determined after fractionation /Da, PS equivalent	Fraction start /min:sec	Fraction end /min:sec
**FR X**	20000	6:09	6:21
**FR IX**	10000	6:36	6:48
**FR VIII**	7000	6:53	7:04
**FR VII**	5000	7:04	7:15
**FR VI**	3000	7:22	7:34
**FR V**	2000	7:47	7:59
**FR IV**	1000	8:23	8:35
**FR III**	580	8:43	8:55
**FR II**	370	9:06	9:18
**FR I**	162	9:47	9:59

Figure S2: 1H-NMR spectra of the investigated lignin samples

Solvent: THF-d8. Further experimental parameters are found in the Materials and methods section of the manuscript. The aromatic region used for DOSY measurements is zoomed on in a red frame in each spectrum.

A

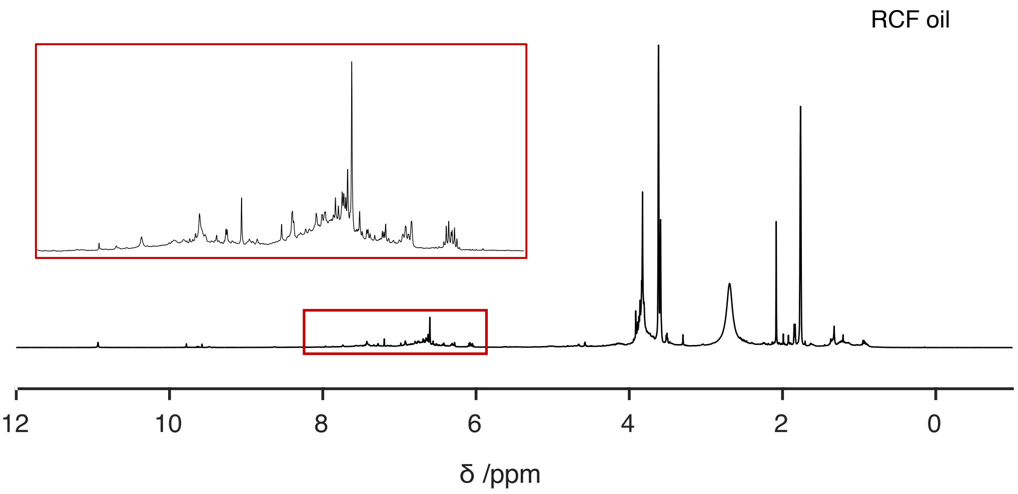

B

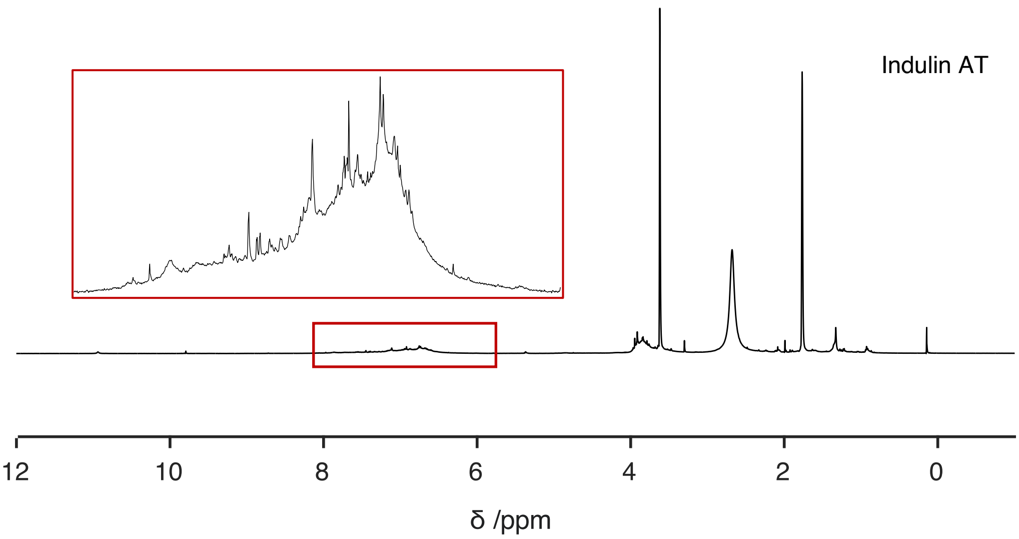

C

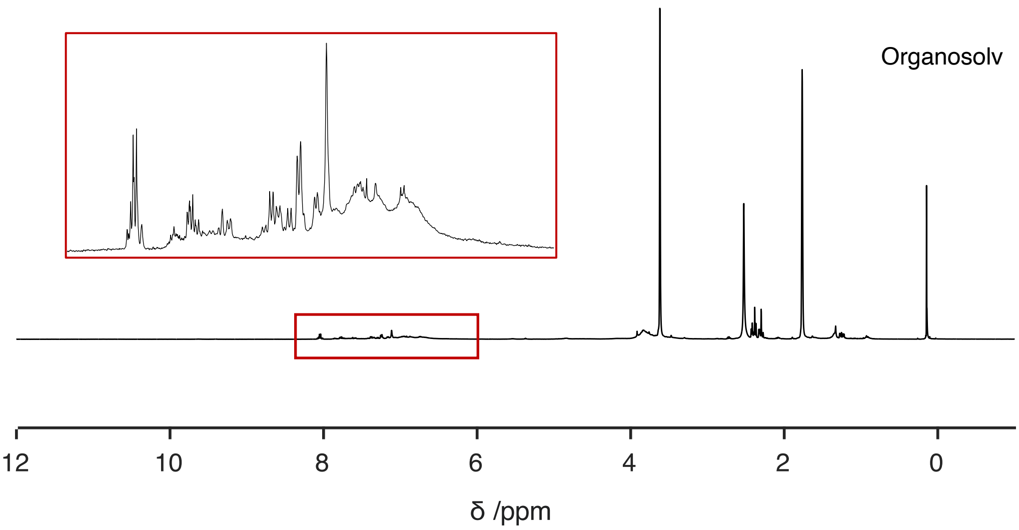

Figure S3: ESI-MS spectrum of dehydrodidihydroeugenol (D1)

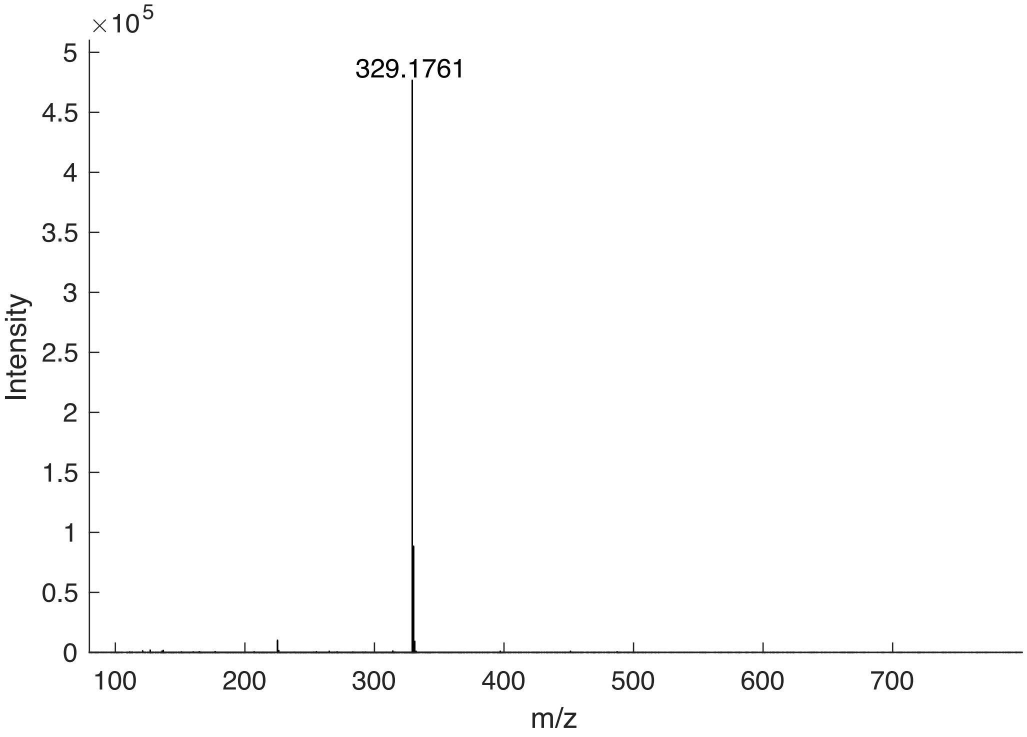

Figure S4: Direct infusion electrospray ionization mass spectra of isolated lignin fractions

Molecular weights shown here were determined directly after fractionation by GPC against PS standards.

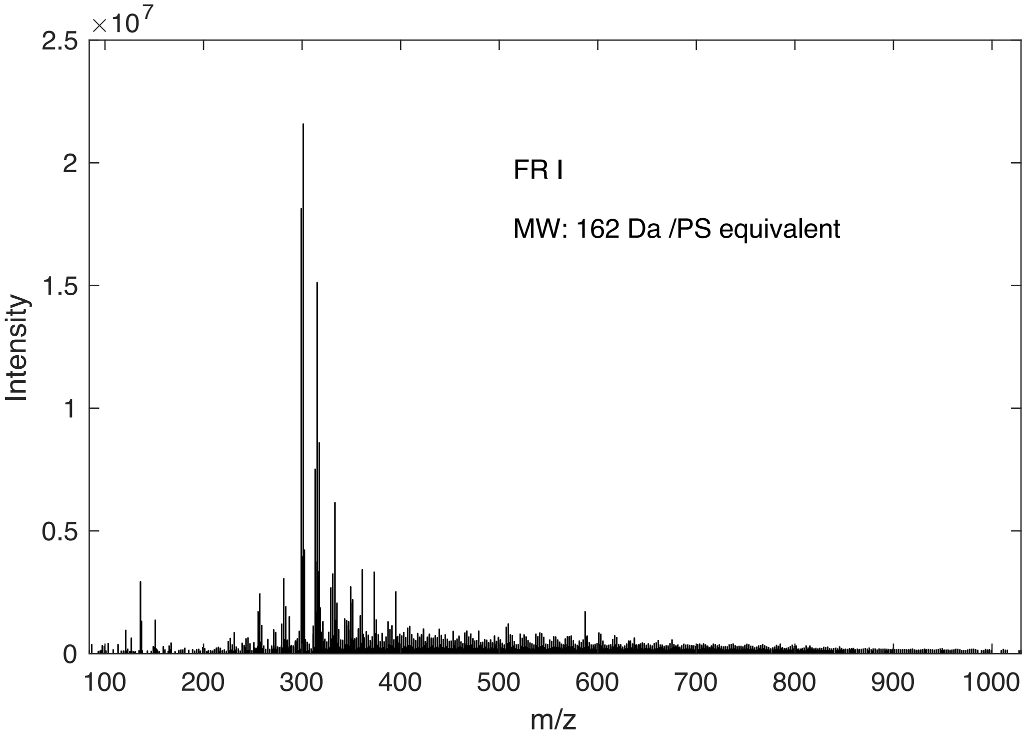

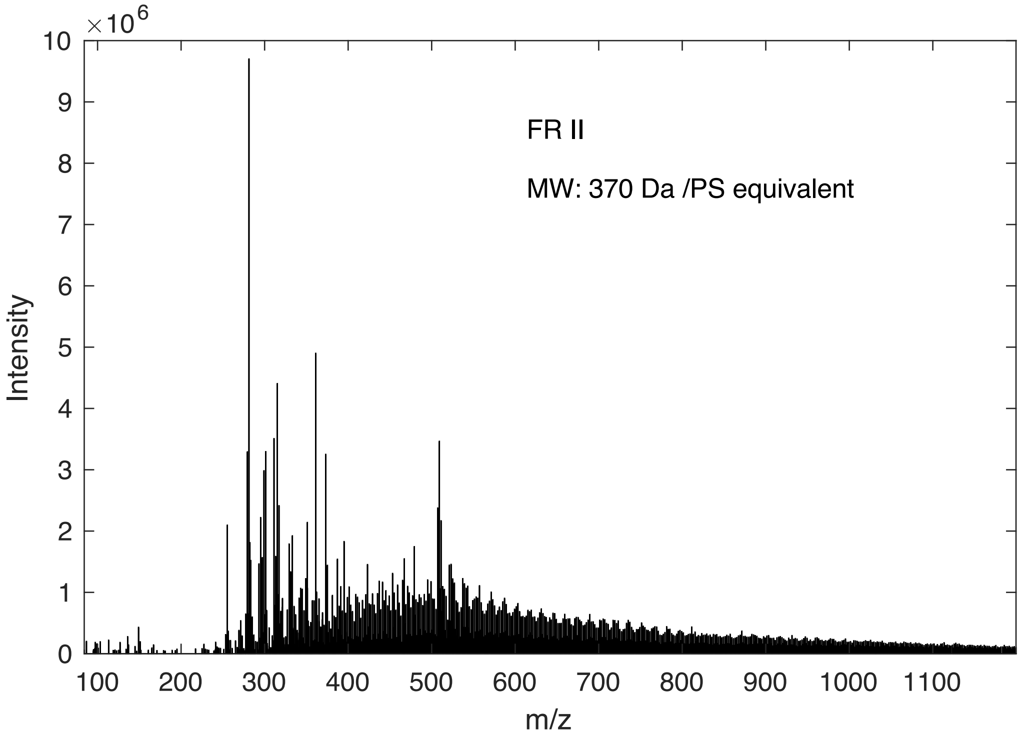

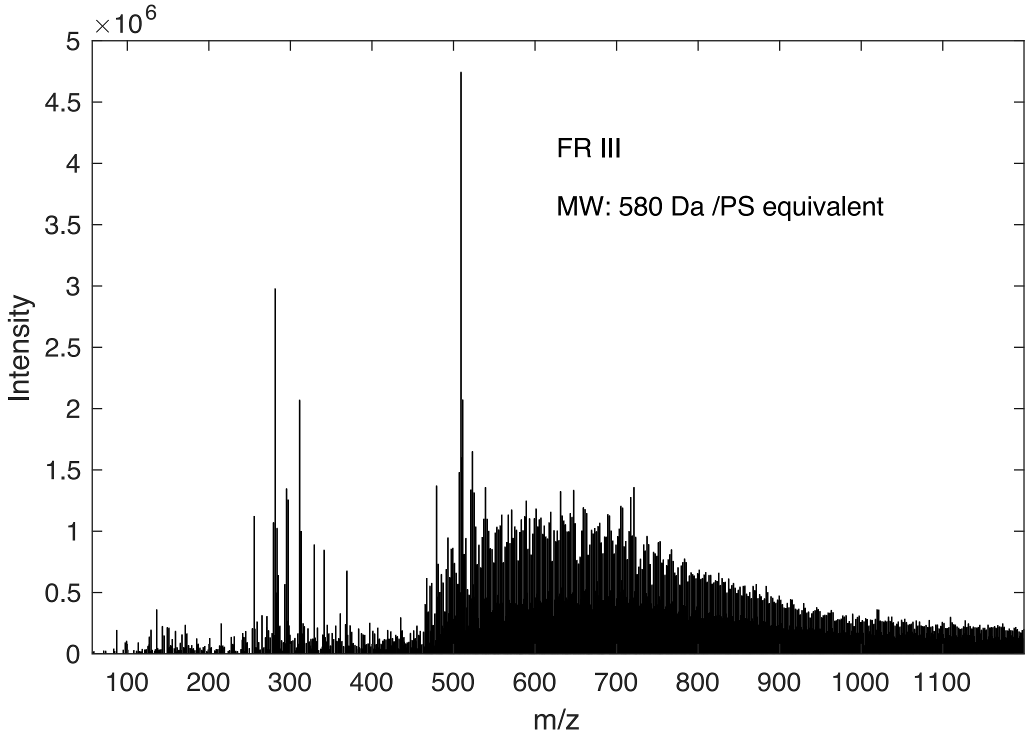
×10^6
FR III
MW: 580 Da /PS equivalent
Intensity
m/z
0
0.5
1
1.5
2
2.5
3
3.5
4
4.5
5
100
200
300
400
500
600
700
800
900
1000
1100

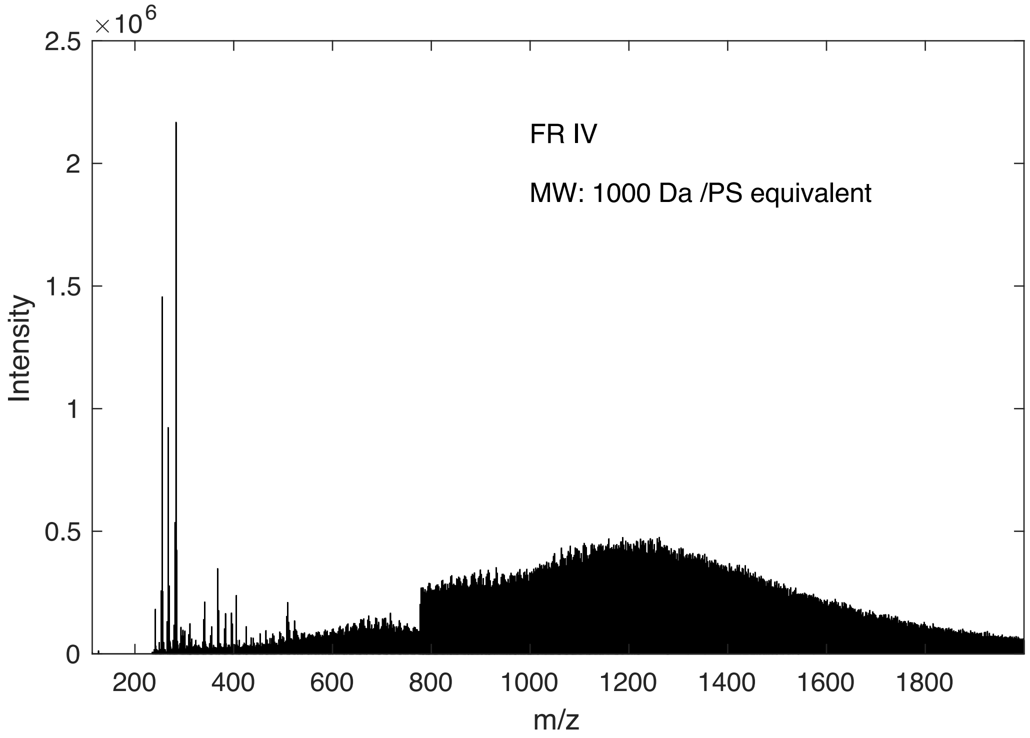
×10^6
FR IV
MW: 1000 Da /PS equivalent
Intensity
m/z
0
0.5
1
1.5
2
2.5
200
400
600
800
1000
1200
1400
1600
1800

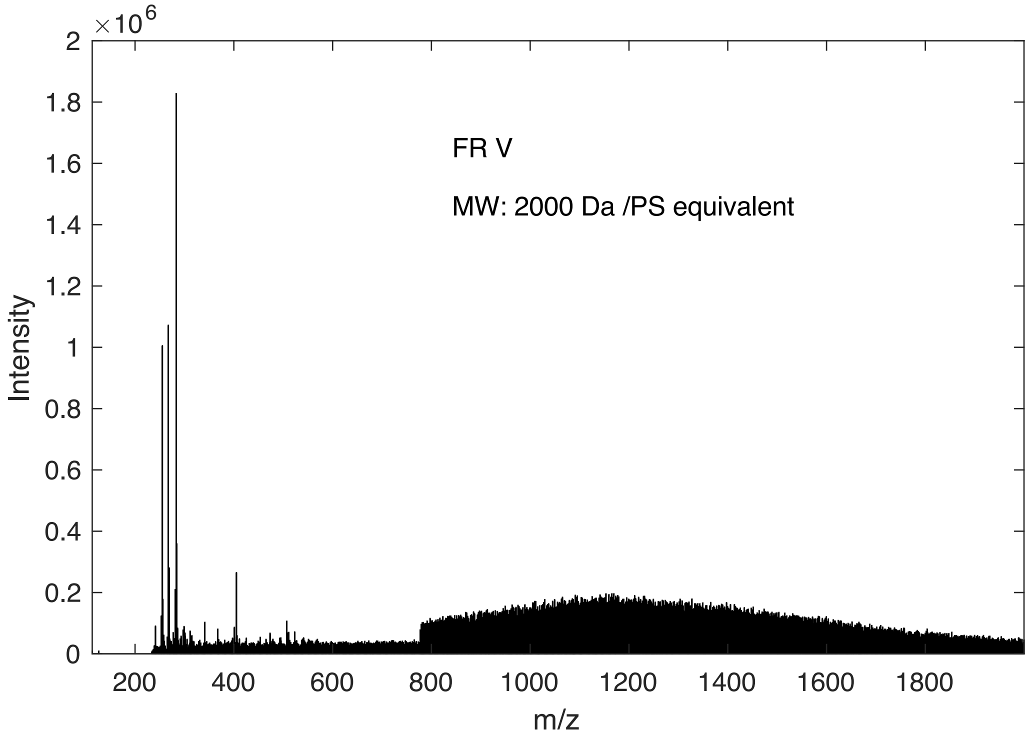
×10^6
FR V
MW: 2000 Da /PS equivalent
Intensity
m/z

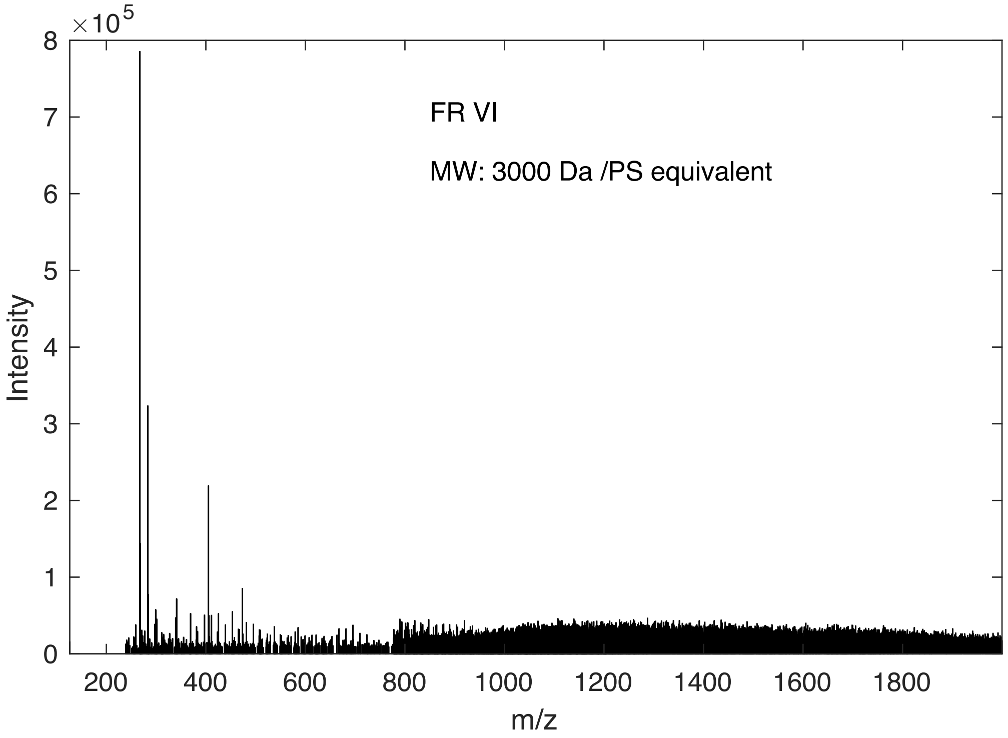
×10^5
FR VI
MW: 3000 Da /PS equivalent
Intensity
m/z

Figure S5: Gel permeation chromatograms of the isolated lignin fractions after one month of storage

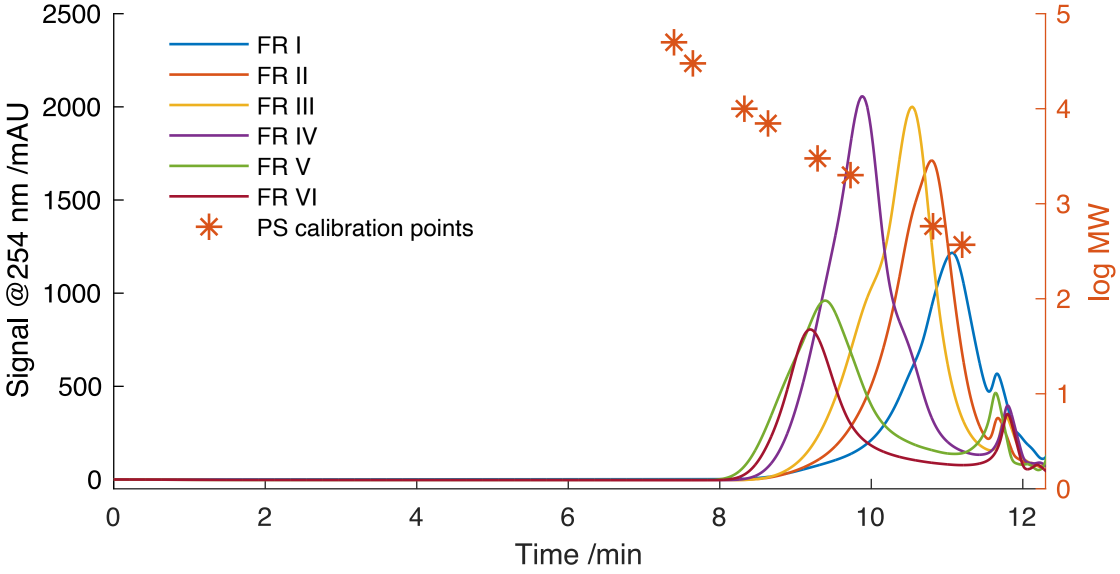

Supplement: Supplementary file 1 — ac4c01187_si_001.pdf [file ac4c01187_si_001.pdf]
